# Community Resource: Large-Scale Proteogenomics to Refine Wheat Genome Annotations

**DOI:** 10.3390/ijms25168614

**Published:** 2024-08-07

**Authors:** Delphine Vincent, Rudi Appels

**Affiliations:** 1 Independent Researcher, Melbourne, VIC 3000, Australia; 2 Faculty of Science, University of Melbourne, Parkville, VIC 3010, Australia; rudi.appels@unimelb.edu.au

**Keywords:** *Triticum aestivum*, bottom-up proteogenomics, proteomics, multiple sequence alignment, genome annotation, gene models

## Abstract

*Triticum aestivum* is an important crop whose reference genome (International Wheat Genome Sequencing Consortium (IWGSC) RefSeq v2.1) offers a valuable resource for understanding wheat genetic structure, improving agronomic traits, and developing new cultivars. A key aspect of gene model annotation is protein-level evidence of gene expression obtained from proteomics studies, followed up by proteogenomics to physically map proteins to the genome. In this research, we have retrieved the largest recent wheat proteomics datasets publicly available and applied the Basic Local Alignment Search Tool (tBLASTn) algorithm to map the 861,759 identified unique peptides against IWGSC RefSeq v2.1. Of the 92,719 hits, 83,015 unique peptides aligned along 33,612 High Confidence (HC) genes, thus validating 31.4% of all wheat HC gene models. Furthermore, 6685 unique peptides were mapped against 3702 Low Confidence (LC) gene models, and we argue that these gene models should be considered for HC status. The remaining 2934 orphan peptides can be used for novel gene discovery, as exemplified here on chromosome 4D. We demonstrated that tBLASTn could not map peptides exhibiting mid-sequence frame shift. We supply all our proteogenomics results, Galaxy workflow and Python code, as well as Browser Extensible Data (BED) files as a resource for the wheat community via the Apollo Jbrowse, and GitHub repositories. Our workflow could be applied to other proteomics datasets to expand this resource with proteins and peptides from biotically and abiotically stressed samples. This would help tease out wheat gene expression under various environmental conditions, both spatially and temporally.

## 1. Introduction

Bread wheat (*Triticum aestivum*) is an allohexaploid species (2n = 6x = 42, AABBDD genomes) resulting from the combination of three interrelated diploid progenitors: *T. urartu* (AA), a relative of *Aegilops speltoides* (BB) and *T. tauschii* (DD) and a major cereal crop widely cultivated across the world [1,2]. It is the staple food of billions of people worldwide and an important source of dietary fiber, proteins, and minerals. In addition, it is widely used in the food and beverage industry, particularly in the production of bread, cereals, pasta, and beer. It is a model for plant domestication and breeding [3]. Wheat is not only economically important but also an essential crop for maintaining global food security. A multilayered global food crisis mitigating near-term food security risks, stabilizing wheat supplies, and transitioning toward long-term agri-food system resilience is proposed to address increasing climate change [4].

The International Wheat Genome Sequencing Consortium (IWGSC) was established in 2005 with the aim of generating a reference genome sequence for *T. aestivum*. After more than a decade of collaborative efforts, IWGSC released the high-quality reference genome sequence of bread wheat in 2018 (IWGSC RefSeq v1.0) [5], which is considered one of the most complex genomes of any crop plant [6]. A chromosome-scale assembly soon followed, locating 2001 previously annotated or unplaced genes and identifying 5799 additional gene copies [7]. Optical mapping and long sequence reads further helped refine the wheat reference genome, leading to the release of IWGSC RefSeq v2.1 in 2020 [8]. This new assembly contains 266,753 genes, including 105,534 High Confidence (HC) genes and 159,840 Low Confidence (LC) genes. The bread wheat reference genome offers a valuable resource for understanding its genetic structure and improving its agronomic traits, particularly resistance to biotic and abiotic stress [6]. Moreover, *T. aestivum* genome sequence provides valuable information for developing new wheat varieties with improved yield, quality, and sustainability [9]. It has also been used in numerous studies, including the elucidation of wheat domestication, evolution, and chromosomal organization [6]. The availability of the IWGSC wheat genome sequence will accelerate breeding efforts aimed at improving wheat productivity and sustainability, and ultimately, help meet growing demand for this important food crop.

Proteogenomics, the interface of proteomics and genomics, has become an active area of research owing to the emergence of new DNA, RNA, and protein sequencing technologies [10]. In this approach, mass spectrometry (MS)-based proteomic data are used to provide protein-level evidence of gene expression and to facilitate the annotation of gene models. There are several approaches to physically map peptides to a genome, and the choice of approach will depend on the available resources, the quality of data, and the research question. One such strategy is bottom-up (BU) proteogenomics which is a data-driven method that involves protein digestion with proteases such as trypsin, peptide separation using high-performance liquid chromatography (HPLC), and peptide sequencing by tandem MS (MS/MS) to identify peptides that map to a genome [11]. BU proteogenomics has several advantages, including high sensitivity, high throughput, and the ability to identify post-translational modifications (PTMs) [12]. However, this approach has some limitations, such as reduced coverage of low-abundance proteins and limited ability to identify large proteins or proteins with extensive sequence variation.

In plants, proteogenomics has improved the sensitivity of protein identification for model and nonmodel species, as well as enabling the analysis of complex genome annotation of polyploid organisms such as sweet potatoes [13]. In hexaploid wheat, a comprehensive BU proteogenomics endeavor on various organs and tissues sampled at key developmental stages was achieved by Duncan et al. in 2017 [14]. As many as 1,457,281 matched spectra, representing 89,754 unique peptides at a 2% false discovery rate (FDR), provided the basis for identifying 15,779 proteins and mapping them along IWGSC RefSeq 1.0. More recently, Vincent et al. extensively analyzed the proteome of more than 4000 harvested and stored wheat grains [15,16] based on 195,426 HPLC-MS/MS spectra and yielding 123,638 mascot hits at 5% FDR corresponding to 14,768 unique peptides assigned to 8738 proteins. As these studies not only represented the most comprehensive wheat proteomics datasets to date but also made their raw data accessible, we retrieved their datasets to apply a BU proteogenomics approach and map peptides against IWGSC RefSeq 2.1 genome. Our objective was to process the raw data to map the peptides to the wheat genome, to analyze the results with suitable analytical and visualization tools, and to make all of our work publicly available.

## 2. Results and Discussion

**Figure 1:**
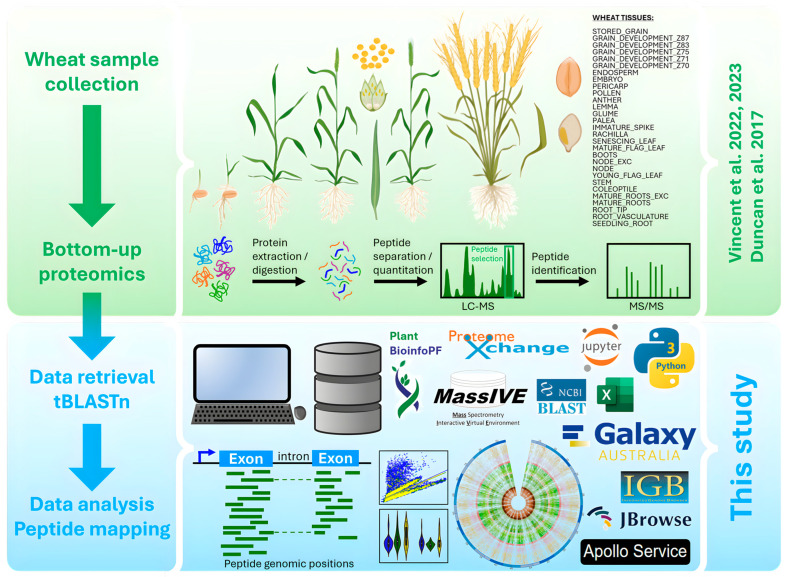
Experimental design of wheat bottom-up (BU) proteogenomics analysis (figure partially created in BioRender). This research was based on organs obtained from plants grown, sampled, and stored in optimal conditions [14,15,16].

This work exploited extensive HPLC-MS/MS proteomics datasets generated by [14,15,16] to physically map identified peptides along *T. aestivum* genome using tBLASTn algorithm and various visualization tools. The experimental design is schematized in Figure 1.

### 2.1. tBLASTn to Align Peptides to Wheat DNA

The algorithm tBLASTn is one of the Basic Local Alignment Search Tool (BLAST) operation modes developed in 1991 [17] that aligns protein (or peptide) sequences to a nucleotide database translated to hypothetical amino acid (AA) sequences in all six reading frames. In 2006, its implementation was modernized with composition-based statistics to greatly improve statistical accuracy and reliability while preserving retrieval accuracy [18]. A tBLASTn search is the only way to pinpoint nucleotide potential coding regions at the protein level. The BLAST+ program was implemented in 2009 to dramatically reduce run times [19]. In 2015, the command line NCBI BLAST+ tool suite was wrapped for use within the Galaxy web-based biomedical data analysis platform [20]. The Galaxy platform offers user-friendly free-to-use tools on generous server space and central processing unit (CPU) times [21]. We took advantage of this resource for our work and ran the sizeable tBLASTn job from Galaxy Australia server (https://usegalaxy.org.au/). 

#### 2.1.1. Optimising the tBLASTn Search 

A critical element in assessing the quality of a pairwise sequence alignment is the scoring matrix that provides a score for aligning any possible pair of residues [4]. Point accepted mutation (PAM) matrices are suitable for short sequence queries [22], while the Blocks substitution matrix (BLOSUM) caters to longer queries [23]. BLOSUM matrices are based on observed alignments, unlike PAM matrices, which are extrapolated from comparisons of closely related proteins. To compare closely related sequences, PAM matrices with lower numbers (e.g., PAM30) or BLOSUM matrices with higher numbers (e.g., BLOSUM90) are typically employed.

Another essential consideration for multiple sequence alignment is modeling insertions and deletions, otherwise known as gaps. The most prevalent model invokes gap open (existence) and gap extension penalties. Varying the gap open and gap extension costs not only results in very different alignments but also yields different distributions of phylogeny scores [24].

By combining the use of BLOSUM or PAM matrices with various gap costs, we performed 12 tests on HC gene *TraesCS4D03G0026600.1* displaying sufficient length (2.2 kb), complexity (5 exons and 4 introns) and peptide coverage (36% with 62 unique peptides). Our test results are summarized in Table 1.

We ranked the methods not only according to the number of correctly aligned peptides but also by whether known gaps were detected or not. Another relevant parameter was CPU time, which varied from 2 to 5 min. Correct alignments ranged from 4% (test 11) to 34% (test 8), and correct gap detection ranged from 0% (tests 2–3, 9–11) to 43% (5, 7–8). The largest number of peptides appropriately aligned (21/62) was achieved with test 8 which applied a PAM-30 matrix with gap costs of existence 10 and extension 1. Moreover, these parameters successfully aligned three peptides with gaps that spanned the first intron (Supplementary Table S1). Additionally, this method took the least amount of time to run (2 min), thus minimizing CPU time when aligning hundreds of thousands of sequences. Consequently, the test 8 method ranked the best and was adopted to align all the peptide sequences from our datasets.

#### 2.1.2. Peptides Mapped by tBLASTn 

Overall, 2,705,657 peptides were recovered from [14,15,16] across 29 tissue types, which listed 45,620 to 153,644 peptides with an average of 93,299 (±32,163) (Table 2). 

As tBLASTn operates on sequence information, we only kept unique AA sequences and eliminated all redundancy related on one hand to charge states and acquired during the electron spray ionization (ESI)-MS analysis, and on the other hand to PTMs, which were abundant [15,16]. A total of 861,759 unique AA peptide sequences were thus searched against the wheat genome (4.2 Gb) using NCBI tBLASTn on the Galaxy Australia server. 

Applying our optimized parameters, the analysis required 11 days to complete the tBLASTn job on Galaxy Australia which allocated us 5 cores and 19.6 GB of memory. The 861,759 AA sequences yielded 92,719 (10.8%) peptide hits. With an average of 10.8% (±7.3), hit success rates ranged from 3.7% (young flag leaf) to 35.9% (stored grain). A total of 46,576 peptides mapped to negative the DNA strand, and 46,143 peptides to the positive DNA strand. The distribution across the 6 reading frames was comparable and averaged 15,453 (±198). All the results are available in Supplementary Table S2.

#### 2.1.3. Peptides Missed by tBLASTn 

An average of 89% of peptides returned no hit. This could be explained by the fact that BLAST program does not search for splice sites or try to distinguish introns from exons, and tBLASTn in particular does not consider the possibility that an alignment could be extended in another reading frame [18].

To test this hypothesis, we randomly chose the HC gene *TraesCS4D03G0026600.1* (2.2 kb) which contains five exons and four introns and exhibits a frame shift. All exons are transcribed into the third reading frame of the positive strand, except exon three, which is transcribed into the second reading frame of the positive strand. 

**Figure 2:**
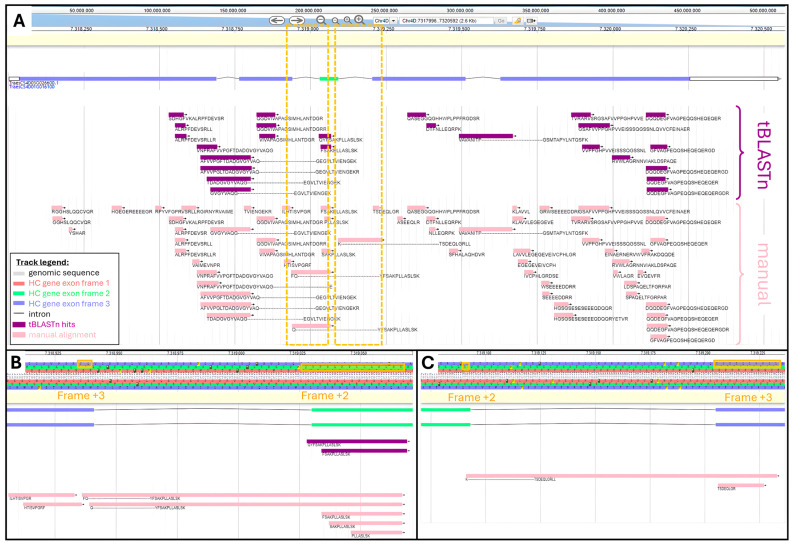
Comparison of tBLASTn and manual alignments along *TraesCS4D03G0026600.1* HC gene viewed in Apollo Jbrowse: (**A**) Alignment along full gene. Boxed areas are zoomed-in in panels (**B**,**C**). tBLASTn hits are purple and manual alignment is pink. (**B**) Zoom-in of genomic region spanning intron between second and third exon. AA sequence is highlighted where frame shift occurs. (**C**) Zoom-in of genomic region spanning intron between 3rd and 4th exon. AA sequence is highlighted where frame shift occurs.

From our recovered datasets, 62 unique peptides belonged to the protein encoded by *TraesCS4D03G0026600.1* gene. We manually performed the physical mapping of all these 62 peptides by mathematically converting their AA coordinates into genomic positions. The tBLASTn search yielded 25 (40%) hits out of 62 peptides (Supplementary Table S3). Both manual and tBLASTn alignments were visualized and compared using the bread wheat Apollo Jbrowse server (Figure 2). 

Figure 2A shows areas not covered by tBLASTn hits, namely ⅔ of the 1st exon, ½ of the third exon, and ⅓ of the fifth exon. We looked at the physicochemical features of *TraesCS4D03G0026600.1* peptides to try and explain why they did not produce a tBLASTn hit. It seemed that missed peptides exhibited short length and therefore low molecular weight (MW), extreme values of Grand Average of Hydrophobicity (GRAVY) beyond a range of 0.6 to −2.2, and an aromaticity greater than 0.14 (Supplementary Table S3). 

TBLASTn adequately aligned peptides beginning in one exon and finishing in the subsequent exon, thus spanning an intron. This was illustrated with peptide AFVVPGFTDADGVGYVAQ---GEGVLTVIENGEK spanning the second intron and peptide VAVANITP---GSMTAPYLNTQSFK spanning the 4th intron.

TBLASTn algorithm could successfully assign peptides that fully aligned with distinct reading frames, as exemplified with peptides QYFSAKPLLASLSK and FSAKPLLASLSK both found within the third exon on the second frame, while all the other peptides aligned along the third frame. However, the program failed to map peptides whose AA sequence resulted from a frame shift, meaning part of their sequence aligned with a given frame and the rest aligned with another reading frame. This was the case for peptides FQ---YFSAKPLLASLSK and Q---YFSAKPLLASLSK, which started in exon 2 on frame +3 and finished in exon 3 on frame +2 (Figure 2B). Another example is provided with peptide K---TSDEQLGRLL, which started in exon 3 on frame +2 and finished in exon 4 on frame +3 (Figure 2C).

Perhaps, this study could help developers to further refine the BLAST+ program so that short peptides and peptides covering frame shifts can reliably return hits.

### 2.2. Proteogenomics to Refine Wheat Gene Annotation

Summarized information for mapped peptides can be shown in different tracks on a Circos viewer or a genome browser [12]. Here, we present two genome browsers, a locally installed version of Integrated Genome Browser (IGB) software 10.0.1 [25] and the online public Apollo Jbrowse server (https://bread-wheat-um.genome.edu.au/apollo/49826/jbrowse/) [26], as well as a circular plot using the Circos tool [27] wrapped in Galaxy platform [28].

#### 2.2.1. Physical Mapping of tBLASTn Peptides 

**Figure 3:**
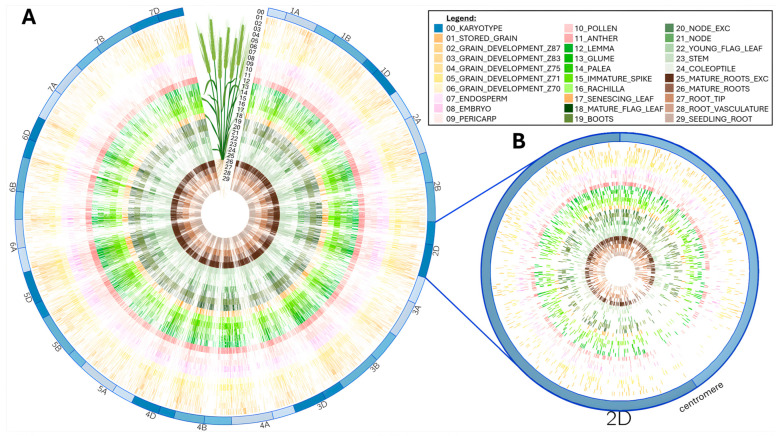
Circos plot of peptides aligned along wheat chromosomes for each tissue (figure partially created in Galaxy Australia): (**A**) Full mapping along all 21 *T. aestivum* chromosomes. (**B**) Zoomed-in view of chromosome 2D to emphasize low peptide alignment around centromeric region.

Circos plots were originally devised to display genome structure [27]. They offer full flexibility to present complex large datasets in a concise and enticing manner. We layered all the mapped peptides according to the wheat tissue they were extracted from [14,15,16] in a circular plot (Figure 3).

This condensed visualization shows that the 92,719 peptides homogenously cover all the chromosomes regardless of tissue type (Figure 3A). Noticeably, centromeric regions present a lesser peptide density coincidently with a reported lack of genes [5]. This can better be appreciated at a single chromosome scale, as exemplified on chromosome 2D in Figure 3B. The wheat proteogenome paucity in the centromere region was previously reported [14,16] and attributed to uneven distribution of coding capacity across the chromosomes [14].

The number of mapped peptides per chromosome varied from 2304 to 8287 with an average of 4411 (Table 3).

Duncan et al. noted a low peptide coverage on chromosomes 1A, 2A, and 4B [14], which was not substantiated by our analysis. In our study, chromosomes 1A, 2A, and 4B featured 3197, 5125, and 4449 peptides, respectively. Chromosomes with the lowest peptide density were 7B (2304), 3D (2859), and 6D (3074), while the most densely covered chromosomes were 5D (5804), 2D (6208), and 3B (8287). There was a strong positive correlation (R^2^ = 0.93) between the number of genes per chromosome and the number of peptides mapped, which is in agreement with chromosome size (Supplementary Figure S1). This confirmed that the variation in peptide density per chromosome matched that of genes. 

Genome browsers such as Integrated Genome Browser (IGB) afford a scalable view of the proteogenomic mapping by allowing general viewing at a whole chromosome level (Supplementary Figure S2A) or very fine detailing by zooming in all the way to the peptide sequence level and ultimately individual nucleotides (Supplementary Figure S2B–D). We can see in Supplementary Figure S2C how processing multiple wheat organs helped increase genome coverage. Indeed, proteogenomic coverage achieved up to 21% of genes located on chromosome 3B and averaged 14% per chromosome (Table 3).

#### 2.2.2. Gene Validation, Promotion and Discovery 

**Figure 4:**
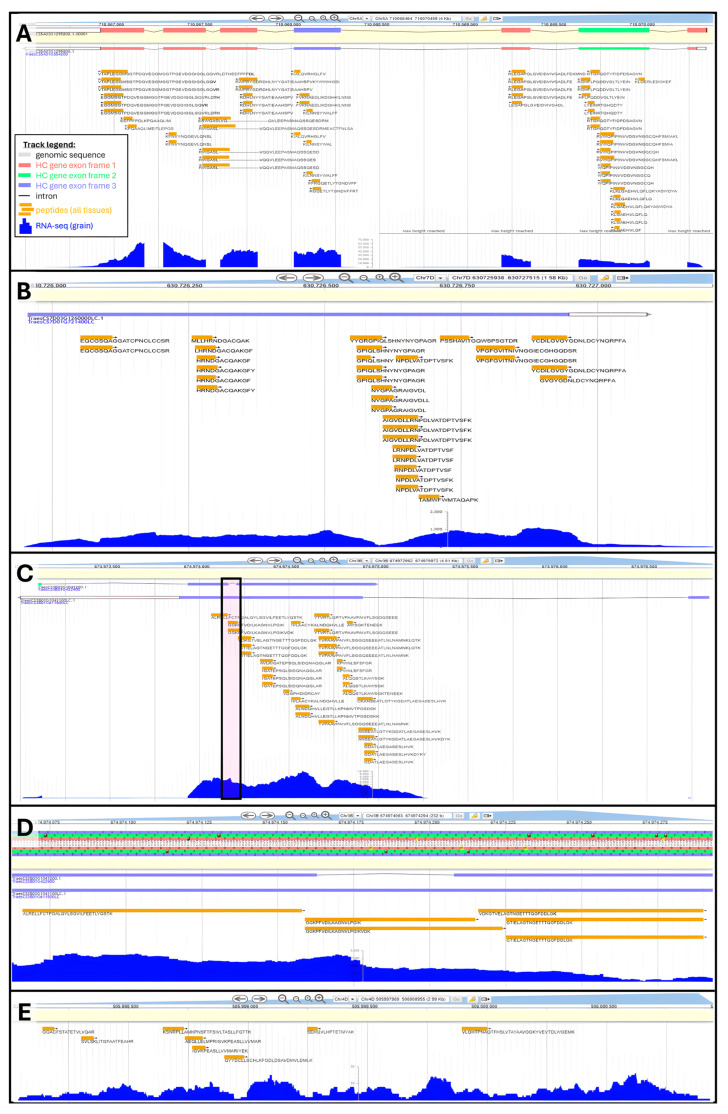
Examples of peptide alignments to refine wheat genome annotation using Apollo Jbrowse Australia: (**A**) Validation of HC gene *TraesCS5A03G451700*. Inset shows track legend across all panels. (**B**) Promotion of LC gene *TraesCS7D03G1260000LC* to HC status. (**C**) Promotion of LC gene *TraesCS3B03G1041100LC* to HC status and amendment of underlying HC gene *TraesCS3B03G1041200*. Boxed area is zoomed-in on Panel (**D**). Panel (**C**) zoomed-in on intron. (**E**) Novel gene discovery exemplified at genomic position Chr4D:505997969..506000955.

Peptide genomic coordinates were used to retrieve the names of HC and LC genes encompassing them. Orphan peptides that could not be assigned a gene were deemed “novel”. Overall, 83,015 unique peptides aligned along 33,612 HC genes thus validating 31.4% of all HC gene models (Table 3). On average, 32% and up to 46% (Chromosome 3B) of HC gene annotations were confirmed by our results. We illustrated annotation validation by proteomics using HC gene *TraesCS5A03G451700* displaying ample peptide sequence coverage along all coding areas (Figure 4A). A few peptides spanned the second intronic region, while the rest aligned with exons translated in reading frames +1, +2, or +3.

A total of 6685 unique peptides were mapped against 3702 LC gene models, which should be promoted to HC status. Overall, 2.3% of all LC annotations were sanctioned by our results (Table 3), reaching 5% on chromosome 3B. We chose the LC gene *TraesCS7D03G1260000LC* to illustrate a proteogenome alignment (Figure 4B). This gene does not feature intronic areas and is well covered by the tBLASTn hits, particularly the second half of its sequence. In another instance, we raise a situation where peptide mapping not only supports the existence of an LC gene but also could be used to refine the annotation of an underlying HC gene. Figure 4C focuses on LC gene *TraesCS3B03G1041100LC* bearing a long exon followed by a much shorter exon. Peptide coverage was extensive along the 1st exon. At these genomic coordinates, HC gene *TraesCS3B03G1041200* displaying a very short intron was also found, which is not endorsed by our mapping results, as peptides GGKPFVDILKAGNVLPGIK and GGKPFVDILKAGNVLPGIKVDK fully span this area and present no gap (Figure 4D). Furthermore, the intron was also marked by an abundant transcript expression. Therefore, HC gene *TraesCS3B03G1041200* deserved to be reannotated. Similar examples can be found in this mapping result, and we hope the wheat community will explore our data and take the required steps to update genome annotations accordingly.

Finally, 2934 peptides could not be assigned any gene; we refer to these orphans as novel peptides. These novel peptides can be used to discover novel *T. aestivum* genes. We highlight this type of scenario on chromosome 4D genomic area 505997969 to 506000955, which featured eight unique peptides and sufficient levels of transcripts to presume the existence of a gene (Figure 4E). 

In all, 37,314 HC and LC gene models were mapped and thus validated in our study. Duncan et al. employed a trypsin-based BU proteomics workflow and identified 15,779 wheat proteins across tissue_nb 3-29 [14]. Vincent et al. identified 8738 proteins from a few stored mature grains following a multiprotease digestion BU proteomics strategy [15] and, later on, screened 4061 stored grain samples using trypsin digestion, which yielded 8044 protein identities [16]. The present study successfully converted protein identities into genomic physical mapping. Our proteogenomic results should be used to update *T. aestivum* genome annotations.

### 2.3. Data Analysis of Mapped Peptides

The outputs from tBLASTn were quantitative thus lending themselves to statistics for identifying trends in alignment processes, which we handled using Python 3 Matplotlib and Seaborn packages extensive statistical and plotting capabilities.

#### 2.3.1. Global Data Analysis 

Global summary statistics showed that mapped peptides contained 8 to 108 AA residues (Supplementary Table S4). The tBLASTn score ranged from 61 to 353, and the percentage of identical matches (pident) varied from 30–100%, containing up to 20 mismatches, a maximum of three gap openings (gapopens), with the longest gap spanning 69 AAs (207 bp). A valuable statistics to assess the alignment confidence is the expectation value (e-value), which indicates the expected number of times the score would occur by chance [29]. Although e-value depends on database size, alignments with expectation values inferior to 0.001 can reliably be used to infer homology. In our study, e-values ranged from 0.01 to 6.03 × 10^−42^ with a Q1 of 2.8 × 10^−11^, Q2 of 6.6 × 10^−5^, and Q3 of 1.8 × 10^−4^. Such e-values placed our hits as true homologs (e-value < 10 × 10^−6^) or closely related sequences (10 × 10^−10^ < e-value < 10 × 10^−50^). 

**Figure 5:**
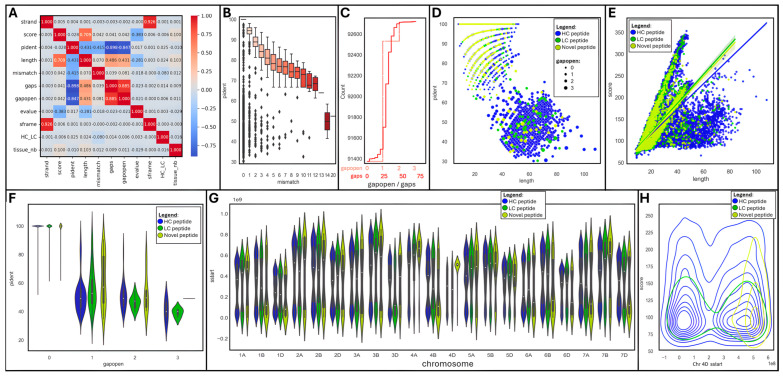
Data visualization of tBLASTn outputs using JupyterLab Python 3 Seaborn and Matplotlib libraries: (**A**) Correlation matrix. (**B**) Boxplot of mismatch vs. pident. (**C**) Cumulative histograms of gaps and gapopens. (**D**) Scatter plot of length vs. pident per peptide type and gapopen size. (**E**) Lm plot of length vs. score per peptide type. (**F**) Violin plot of gapopen vs. pident per peptide type. (**G**) Violin plot of tissue vs. score. (**H**) Density plot of chromosome 4D sstart vs. score per peptide type.

We produced a correlation matrix on a subset of numerical variables to observe which ones were associated (Figure 5A). 

Unsurprisingly, strands and reading frames (sframe) were very strongly positively correlated (R^2^ = 0.926); so were gap openings (gapopens) and sizes (gaps) (R^2^ = 0.885). Yet, they were not correlated with any other variable. Interestingly, peptide length was positively associated with score (R^2^ = 0.709), gap size (R^2^ = 0.486), and openings (R^2^ = 0.431), as well as negatively linked to identity percentage (R^2^ = −0.431) and e-value (R^2^ = −0.281). Moreover, the percentage of identical matches (pident) was strongly negatively correlated with gap size (R^2^ = −0.898) and number (R^2^ = −0.847), and to a lesser extent with the number of mismatches (R^2^ = −0.415). 

A box plot further emphasized that the percentage of identical matches progressively diminished from 100 to 70% as the number of mismatches augmented, dropping down to 50% when mismatches exceeded 13 (Figure 5B). Most mapped peptides were gapless (91,259, 98%); 1254 (1.4%), 197 (0.2%), and 9 peptides hosted 1, 2, or 3 gaps, respectively (Figure 5C). Gap sizes oscillated between 1 and 69, with the greatest frequency from 24–37, thus only spanning short introns.

A pairgrid function enabled us to plot multiple aspects of our tBLASTn dataset in a single chart for a quick appraisal of the main variables (Supplementary Figure S3A). Some clear patterns appeared when peptide types were highlighted (HC, LC, or novel) and were further drilled into. A scatterplot of peptide length versus the percentage of identical matches (pident) highlighted a negative relationship between both variables; most novel peptides achieved high identity despite short sizes (Figure 5D). The introduction of gaps (gapopen) logically increased peptide lengths at the cost of sequence homology. An lmplot of length against score confirmed their strong positive correlation; short novel peptides were clustered and exhibited a wide range of scores (Figure 5E), despite high levels of identical matches (pident) as indicated above. The effect of gap introduction on sequence homology can be seen on a violin plot featuring gapopens versus pident, with a drastic drop in the percentage of identical matches as soon as a gap exists (Figure 5F). 

Another violin plot showing the distribution of peptide starting position (sstart) per chromosome revealed a fairly homogenous pattern across all chromosomes regardless of peptide types (HC, LC, or novel) (Figure 5G), with the exception of chromosome 4D, which presented unusually high density of novel peptides towards its end tail. 

#### 2.3.2. Focus on Chromosome 4D 

Looking in more detail at chromosome 4D via a distplot of peptide starting position (sstart) against scores further illustrated the conglomeration of novel peptides within the terminal region of the chromosome (Figure 5H). HC and LC peptides were homogenously distributed along the whole chromosome. 

Visualizing the last 5.6 Mb of chromosome 4D in Apollo Jbrowse showed 143 novel peptides in this gene-depleted zone (Supplementary Figure S4A). By zooming into regions where transcripts are abundant, such as the 3.6 Kb area covering 515,319,968 to 515,323,561 and mapping 20 peptides, we can presume the existence of 1 or 2 candidate genes (Supplementary Figure S4B). Other likely candidate genes are outlined in regions 515873999–515890302 (16.3 Kb Supplementary Figure S4C) and 517010333–517013855 (3.52 Kb Supplementary Figure S4D).

We investigated whether a link existed between novel peptides and wheat tissues. All peptides mapped homogenously along the genome, as shown on the Circos plot (Figure 3) and more explicitly using box plots (Supplementary Figure S3B). When filtering the data to novel peptides only for all chromosomes, tissue differences appeared, as evidenced by the variation in quartiles (Supplementary Figure S3C). Peptide numbers ranged from 35 (young flag leaf) to 352 (mature root exc.). When focusing on novel peptides aligned along chromosome 4D only, the majority of tissues condensed novel peptides in the end-tail region of the chromosome, with the exception of grain developmental stage Z87 more widely distributed (Supplementary Figure S3D). Grain developmental stages Z83 (tissue_nb 3), Z70 (tissue_nb 6), pollen (tissue_nb 10), mature flag leaf (tissue_nb 18), young flag leaf (tissue_nb 22), root tip (tissue_nb 27), and root vasculature (tissue_nb 28) yielded very few novel peptides on chromosome 4D. Therefore, our results suggest that analyzing different organ types helped detect novel peptides. 

## 3. Materials and Methods

The experimental design is schematized in Figure 1. 

### 3.1. Raw Data Retrieval and Processing

#### 3.1.1. Data Source, Conversion, and Redundancy Removal

*T. aestivum* genomic sequence (IWGSC RefSeq v2.1 [8]) was retrieved from https://urgi.versailles.inra.fr/download/iwgsc/IWGSC_RefSeq_Assemblies/v2.1/ (accessed on 25 August 2023) as a fasta file. Genome annotations were downloaded from https://urgi.versailles.inrae.fr/download/iwgsc/IWGSC_RefSeq_Annotations/v2.1/ (accessed on 25 August 2023) for HC and LC genes as gff3 files. 

MS-proteomics outputs of [14,15,16] were accessed from the following public repositories: ProteomeXchange (https://www.proteomexchange.org/, accessed on 11 May 2024) dataset PXD004720 and MassIVE (https://massive.ucsd.edu/ProteoSAFe/static/massive.jsp, accessed on 11 May 2024) datasets MSV000088253 and MSV000090572. An example of LC-MS/MS spectrum is provided (Supplementary Figure S5). These recent studies represented the most extensive proteome coverage of 29 wheat tissues to date (Table 2).

Pep.xml files were downloaded directly into Galaxy Australia platform [21] version 24.1.2 using the “Upload data” and “Paste/Fetch data” tools and pasting the relevant FTP URLs. The pep.xml files were converted into tab-delimited text files using the “PepXML to Table” tool.

Tabular outputs were exported to MS Office 365 Excel. Decoy peptides were discarded and an index column with unique identifiers was added for tracking purposes. Redundant target peptide AA sequences were eliminated using the “Remove Duplicates” tool. Nonredundant peptides from each tissue were combined into a single table using the “Get Data From Folder” tool and were formatted into a fasta file using the index and the peptide AA sequence.

#### 3.1.2. Database Creation and tBLASTn Search

Both peptide and wheat DNA fasta files were imported into Galaxy Australia platform [21] version 24.1.2 using the “Upload data” and “Choose local file” tools. 

The genomic fasta file was converted into a BLAST database using the “NCBI BLAST+ makeblastdb” tool.

Several tBLASTn substitution matrices and gap costs were tested on test gene *TraesCS4D03G0026600.1*: BLOSUM45, BLOSUM90, PAM30, PAM250, with 5–21 existences and 1–3 extensions. Results are summarized in Table 1 and Supplementary Table S1. The tBLASTn search was performed on all unique peptide sequences against wheat genome using the “NCBI BLAST+ tblastn” tool [19,20] with the following optimized parameters: peptide fasta sequence as the query sequences, DNA blastdbn file as the nucleotide BLAST database, traditional tblastn as BLAST type, 0.01 expectation value cutoff, extended 25 columns tabular output, standard genetic code, PAM30 scoring matrix with gap costs existence 10 and extension 1, 3 maximum hits, 1 maximum HSP, 25% minimum coverage, and default-composition-based statistics.

The tBLASTn output was exported to be further processed in Excel and Python 3 and is available in Supplementary Table S2.

### 3.2. Peptide Mapping and Data Analysis

#### 3.2.1. Gene Assignment

Wheat genome gff3 annotations were parsed in miniconda JupyterLab Python 3 (version 3.6.3) to extract gene names, chromosome names, LC or HC status, and gene start and end positions and saved as a CSV file. A Python 3 script was written to loop through each tBLASTn hit and, where possible, assign it to a gene if the chromosome names matched, the peptide start position was greater than the gene start position, and the peptide end position was smaller than the gene end position. Peptides that were not assigned to a gene were deemed “novel”.

#### 3.2.2. Peptide Physical Mapping Using Genome Browsers

Relevant columns from the tBLASTn output (saccver, sstart, send, qseq, score, strand) were extracted to create BED files for each individual tissue, as well as combined into one single file. BED files were permanently uploaded into the bread wheat Apollo Jbrowse server (uploaded on 21 June 2024 at https://bread-wheat-um.genome.edu.au/apollo/49826/jbrowse/) [26] under the label “Proteome hit (tBlastn)”.

The genomic sequence, GFF3, and BED files were also uploaded into a locally installed Integrated Genome Browser (IGB) [25] (https://www.bioviz.org/, accessed on 15 May 2024) for further visualization.

#### 3.2.3. Peptide Physical Mapping Using Circos 

Relevant columns from the tBLASTn output (saccver, sstart, send) were extracted to create TXT files for each individual tissue. The wheat karyotype TXT file was created by retrieving the genomic start and end positions of each chromosome. Centromere positions were obtained from [7].

The files were imported into Galaxy Australia platform [21] using the “Upload data” and “Choose local file” tools. The circular plot was produced using the “Circos” tool [27,28] and each tissue as a 2D Data Plot and plot types set to “Highlight”. 

#### 3.2.4. Mathematical Mapping of Peptides against *TraesCS4D03G0026600.1* Gene 

The 62 mascot-identified peptides obtained from [15,16] and assigned to HC gene *TraesCS4D03G0026600.1* were retrieved along with their start and end position in the protein AA sequence. The gene structure was extracted from GFF3 file and the positions of the 5 exons and 4 introns were computed in miniconda JupyterLab Python 3. A Python script was written to loop through each peptide and mathematically convert the AA coordinates into nucleotide coordinates by considering the genomic position and size of exons and introns. The output was exported as CSV and BED files.

The tBLASTn peptide hits assigned to *TraesCS4D03G0026600.1* gene were extracted and saved as CSV and BED files.

Both BED files were uploaded into the bread wheat Apollo Jbrowse server for comparison purposes. Both CSV files were combined into Supplementary Table S3. 

Various physicochemical parameters for *TraesCS4D03G0026600.1* peptides were computed based on AA squences using BioPython SeqUtils ProtParam module: length, MW, GRAVY, aromaticity, and isoelectric point (pI).

#### 3.2.5. Data Analysis, Statistics, and Visualization

The tBLASTn output was imported into miniconda JupyterLab Python 3 (version 3.6.3) and converted into a pandas dataframe. Descriptive statistics were produced using the pandas method “.describe()” and consigned to Supplementary Table S4.

Correlation matrix and all charts (boxplot, violin plot, lmplot, scatterplot, hitsplot distplot, pairgrid, and joinplot) were generated using matplotlib and seaborn libraries. 

Our Jupyter Notebook Python code is publicly available via GitHub (uploaded on 4 August 2024 at https://github.com/dlf2024/Python_Wheat_Proteogenomics).

## 4. Conclusions

To our knowledge, this is the largest proteogenomics study tackled in a single experiment. In this work, we optimized tBLASTn parameters to align 861,759 unique peptides along IWGSC RefSeq 2.1 genome. Of the 92,719 hits, 89,785 (97%) confirmed the existence of 37,314 HC and LC gene models. Of these, 83,015 unique peptides aligned along 33,612 HC genes, thus validating 31.4% of all HC gene models. Additionally, 6685 unique peptides mapped against 3702 LC gene models (2.3% of all LC annotations), thus deserving HC promotion. The remaining 2934 novel peptides should be used for gene discovery. We supply all our results as a resource for the wheat community, and we hope that IWGSC will use our data to refine *T. aestivum* genome annotation.

A large proportion of peptides (89%) did not produce a hit, among those were peptides exhibiting a reading frame shift mid-sequence. We urge developers to further improve BLAST+ program so that short peptides and peptides covering frame shifts can reliably return hits. We provide a relatively simple workflow that can be applied to any other BU proteomics datasets and hopefully expand this resource with proteins and peptides from biotically and abiotically stressed samples. 

## Figures and Tables

**Table 1:** Summary of tBLASTn tests on *TraesCS4D03G0026600.1* gene.

Test No.	Scoring Matrix	Type	Existence	Extension	CPU ^1^ Time (min)	Total Peptides	Wrong Peptides	Not Found	Correct Peptides	Correct Alignment (%)	Total Gaps	Not Found	Correct Gaps	Correct Gaps (%)	Rank
8	PAM	30	10	1	2	62	1	40	21	33.9	7	4	3	42.9	1
5	BLOSUM	90	11	1	3	62	0	43	19	30.6	7	4	3	42.9	2
7	PAM	30	8	1	2	62	0	44	18	29.0	7	4	3	42.9	3
4	BLOSUM	90	9	1	4	62	0	45	17	27.4	7	5	2	28.6	4
6	PAM	30	5	2	3	62	1	44	17	27.4	7	5	2	28.6	4
1	BLOSUM	90	6	2	4	62	1	45	16	25.8	7	6	1	14.3	6
9	PAM	30	14	2	2	62	3	39	20	32.3	7	7	0	0.0	7
10	PAM	30	15	3	4	62	3	39	20	32.3	7	7	0	0.0	7
3	BLOSUM	90	9	2	2	62	1	43	18	29.0	7	7	0	0.0	9
2	BLOSUM	90	8	2	4	62	1	44	17	27.4	7	7	0	0.0	10
12	BLOSUM	45	19	1	2	62	2	46	14	22.6	7	6	1	14.3	11
11	PAM	250	21	1	5	62	0	59	3	4.8	7	7	0	0.0	12

^1^ CPU, central processing unit.

**Table 2:** Number of peptides processed per tissue type and summary statistics.

Tissue Type	Tissue Number	Total Peptides	Unique AA ^1^ Sequences	tBLASTn ^2^ Hits	tBLASTn ^2^ Hits (%)
STORED GRAIN	1	123,638	14,768	5302	35.9
GRAIN DEVELOPMENT Z87	2	79,826	25,855	1915	7.4
GRAIN DEVELOPMENT Z83	3	84,356	26,818	1925	7.2
GRAIN DEVELOPMENT Z75	4	84,112	28,860	2298	8.0
GRAIN DEVELOPMENT Z71	5	122,739	38,790	3399	8.8
GRAIN DEVELOPMENT Z70	6	90,963	27,402	2579	9.4
ENDOSPERM	7	71,182	22,051	2389	10.8
EMBRYO	8	56,489	25,575	2154	8.4
PERICARP	9	102,924	30,070	3206	10.7
POLLEN	10	51,251	14,180	974	6.9
ANTHER	11	138,308	36,539	9743	26.7
LEMMA	12	119,032	30,885	2443	7.9
GLUME	13	131,255	30,545	3566	11.7
PALEA	14	92,451	25,637	3037	11.8
IMMATURE SPIKE	15	132,013	39,797	5547	13.9
RACHILLA	16	130,559	35,184	2346	6.7
SENESCING LEAF	17	61,147	21,388	1290	6.0
MATURE FLAG LEAF	18	78,745	31,959	1974	6.2
BOOTS	19	61,154	29,800	5167	17.3
NODE EXC	20	83,310	35,434	1384	3.9
NODE	21	73,460	27,589	2050	7.4
YOUNG FLAG LEAF	22	59,966	25,438	948	3.7
STEM	23	49,383	21,945	1687	7.7
COLEOPTILE	24	153,644	45,500	5839	12.8
MATURE ROOTS EXC	25	45,620	34,308	9357	27.3
MATURE ROOTS	26	89,494	32,528	2759	8.5
ROOT TIP	27	136,241	39,486	3140	8.0
ROOT VASCULATURE	28	66,587	21,877	1285	5.9
SEEDLING ROOT	29	135,808	41,551	3016	7.3
	**SUM**	2,705,657	861,759	92,719	10.76
	**MIN**	45,620	14,180	948	3.73
	**MAX**	153,644	45,500	9743	35.90
	**AVERAGE**	93,299	29,716	3197	10.83
	**SD**	32,163	7594	2197	7.34

^1^ AA, amino acid; ^2^ BLAST, Basic Local Alignment Search Tool.

**Table 3:** Number of peptides and genes aligned along wheat chromosomes.

Chromosome	HC ^1^ Peptides	LC ^2^ Peptides	Novel Peptides	SUM ^3^ Peptides	HC ^1^ Pep-Mapped Genes	LC ^2^ Pep-Mapped Genes	SUM ^3^ Pep-Mapped Genes	All HC ^1^ Genes	All LC ^2^ Genes	SUM All Genes	HC ^1^ %	LC ^2^ %	SUM ^3^ %	Chro_Size ^4^
Chr1A	2883	235	79	3197	1286	128	1414	4359	6509	10,868	29.5	2.0	13.0	594,442,527
Chr1B	3957	296	154	4407	1515	173	1688	4736	8112	12,848	32.0	2.1	13.1	700,547,350
Chr1D	3332	224	67	3623	1329	114	1443	4487	6006	10,493	29.6	1.9	13.8	498,638,509
Chr2A	4599	322	204	5125	1950	197	2147	5840	7884	13,724	33.4	2.5	15.6	787,782,082
Chr2B	4523	448	207	5178	1994	255	2249	6152	9631	15,783	32.4	2.6	14.2	812,755,788
Chr2D	5739	332	137	6208	2137	181	2318	5885	7550	13,435	36.3	2.4	17.3	656,544,405
Chr3A	3095	231	87	3413	1391	141	1532	5237	7572	12,809	26.6	1.9	12.0	754,128,162
Chr3B	6971	849	467	8287	2739	467	3206	5941	9351	15,292	46.1	5.0	21.0	851,934,019
Chr3D	2589	207	63	2859	1194	103	1297	5306	6726	12,032	22.5	1.5	10.8	619,618,552
Chr4A	4113	327	130	4570	1641	176	1817	4870	7680	12,550	33.7	2.3	14.5	754,227,511
Chr4B	4048	318	83	4449	1490	171	1661	3878	6324	10,202	38.4	2.7	16.3	673,810,255
Chr4D	3848	277	274	4399	1447	122	1569	3582	4870	8452	40.4	2.5	18.6	518,332,611
Chr5A	3464	225	64	3753	1353	145	1498	5450	7604	13,054	24.8	1.9	11.5	713,360,525
Chr5B	4752	443	117	5312	1942	212	2154	5574	8288	13,862	34.8	2.6	15.5	714,805,278
Chr5D	5327	372	105	5804	1983	179	2162	5574	6803	12,377	35.6	2.6	17.5	569,951,140
Chr6A	3631	287	152	4070	1450	164	1614	4141	6377	10,518	35.0	2.6	15.3	622,669,697
Chr6B	3423	283	106	3812	1326	164	1490	4627	8433	13,060	28.7	1.9	11.4	731,188,232
Chr6D	2802	196	76	3074	1272	134	1406	4012	5318	9330	31.7	2.5	15.1	495,380,293
Chr7A	3052	236	124	3412	1339	142	1481	5573	8324	13,897	24.0	1.7	10.7	744,491,536
Chr7B	1991	212	101	2304	956	138	1094	4892	8602	13,494	19.5	1.6	8.1	764,081,788
Chr7D	4876	365	137	5378	1866	190	2056	5419	7666	13,085	34.4	2.5	15.7	642,921,167
ChrUn *	64	21	0	85	12	6	18	1379	4216	5595	0.9	0.1	0.3	351,582,993
**SUM**	83,079	6706	2934	92,719	33,612	3702	37,314	106,914	159,846	266,760	31.4	2.3	14.0	340,075
**MIN**	1991	196	63	2304	956	103	1094	3582	4870	8452	20	2	8	495,380,293
**MAX**	6971	849	467	8287	2739	467	3206	6152	9631	15,783	46	5	21	851,934,019
**AVERAGE**	3953	318	140	4411	1600	176	1776	5025	7411	12,436	32	2	14	677,219,592

^1^ HC, High Confidence; ^2^ LC, Low Confidence; ^3^ SUM, summation; ^4^ Chro_size, size, or wheat chromosome; * Only peptides from stored grains [16] could be mapped to ChrUn, which is considered here as an outlier and therefore not used in the data analysis.

## Data Availability

All the results reported in this study are available as Supplementary Files, including all peptide mapping BED files for upload in IGB or *T. aestivum* Apollo Jbrowse repository (https://bread-wheat-um.genome.edu.au/apollo/49826/jbrowse/), The Python code and Galaxy workflow, as well as BED files are available on GitHub (https://github.com/dlf2024/Python_Wheat_Proteogenomics).

## Supplementary Materials

The supporting information can be downloaded at https://www.mdpi.com/article/10.3390/ijms25168614/s1.

## Abbreviations

AA, amino acid; BED, Browser Extensible Data; BLAST, Basic Local Alignment Search Tool; BLOSUM, BLOcks SUbstitution Matrix; BU, bottom-up; CPU, central processing unit; DNA, deoxyribonucleic acid; e-value, expectation value; ESI, electron spray ionization; FDR, false discovery rate; gapopen, number of gap openings; GRAVY, Grand Average of Hydrophobicity; HC, High Confidence; HPLC, high-performance liquid chromatography; IGB, Integrated Genome Browser; IWGSC, International Wheat Genome Sequencing Consortium; LC, Low Confidence; MS, mass spectrometry; MS/MS, tandem mass spectrometry; MW, molecular weight; PAM, Point Accepted Mutation; pI, isoelectric point; pident, percentage of identical matches; PTM, post-translational modification; RefSeq, reference sequence; RNA, ribonucleic acid; send, end of BLAST alignment in subject (database hit); sframe, reading frame; sstart, start of BLAST alignment in subject (database hit).
