# Supplementary material for: Community Resource: Large-Scale Proteogenomics to Refine Wheat Genome Annotations
Source: Int J Mol Sci. 2024 Aug 7;25(16):8614. doi: 10.3390/ijms25168614 (PMC11354340; doi:10.3390/ijms25168614)

Supplementary Figure S1: Joinplot between number of mapped peptides and wheat genes for each chromosome.

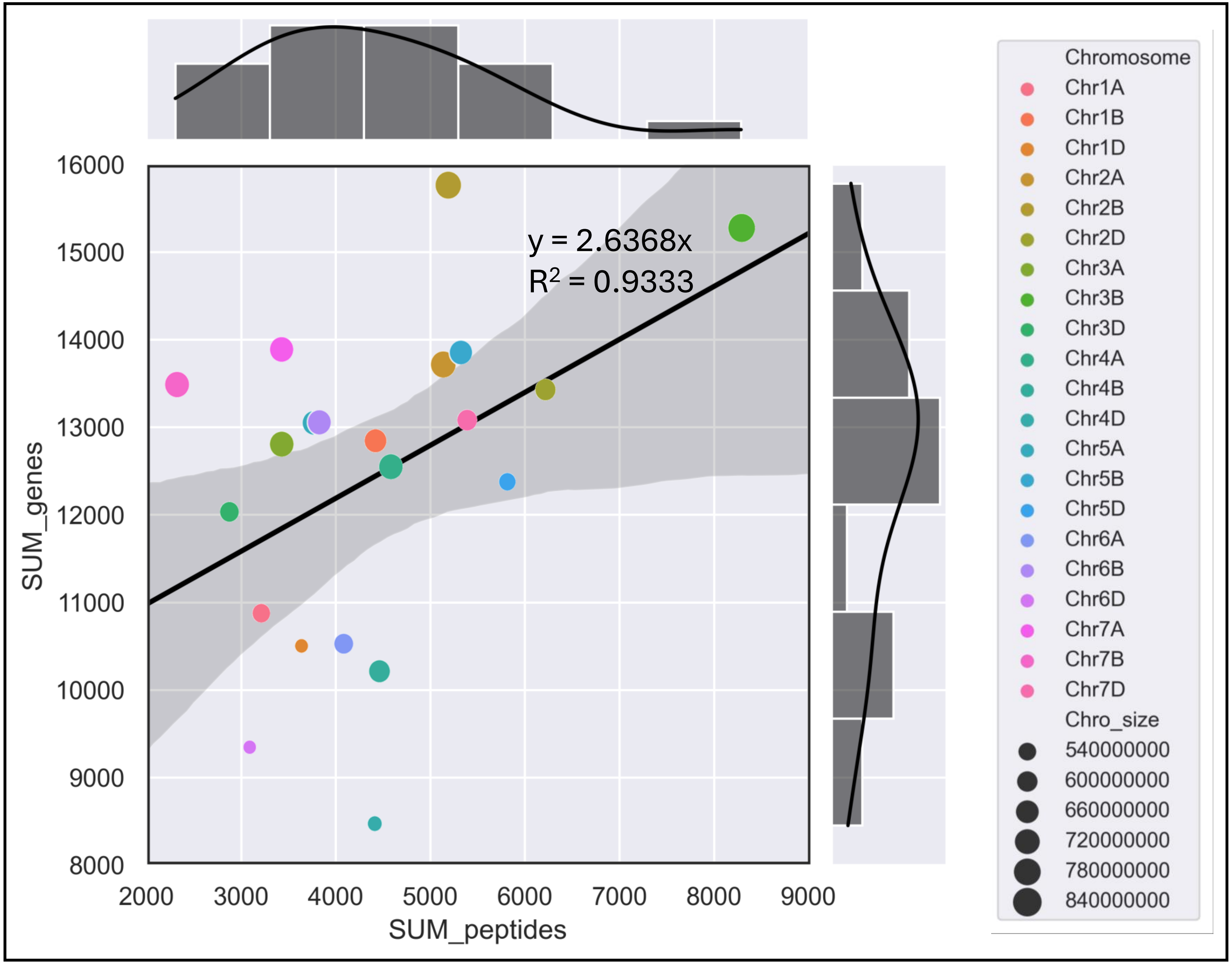

Supplementary Figure S2: Physical mapping of all tBLASTn peptide hits coloured by tissue and viewed in IGB. A. General view of chromosome 2D. Orange dotted line locates gene TraesCS2D03G0133100. B. Zoom in on HC gene TraesCS2D03G0133100. C. Further zoomed in to read peptide AA sequences. D. Even further zoomed in to read DNA nucleotide sequence.

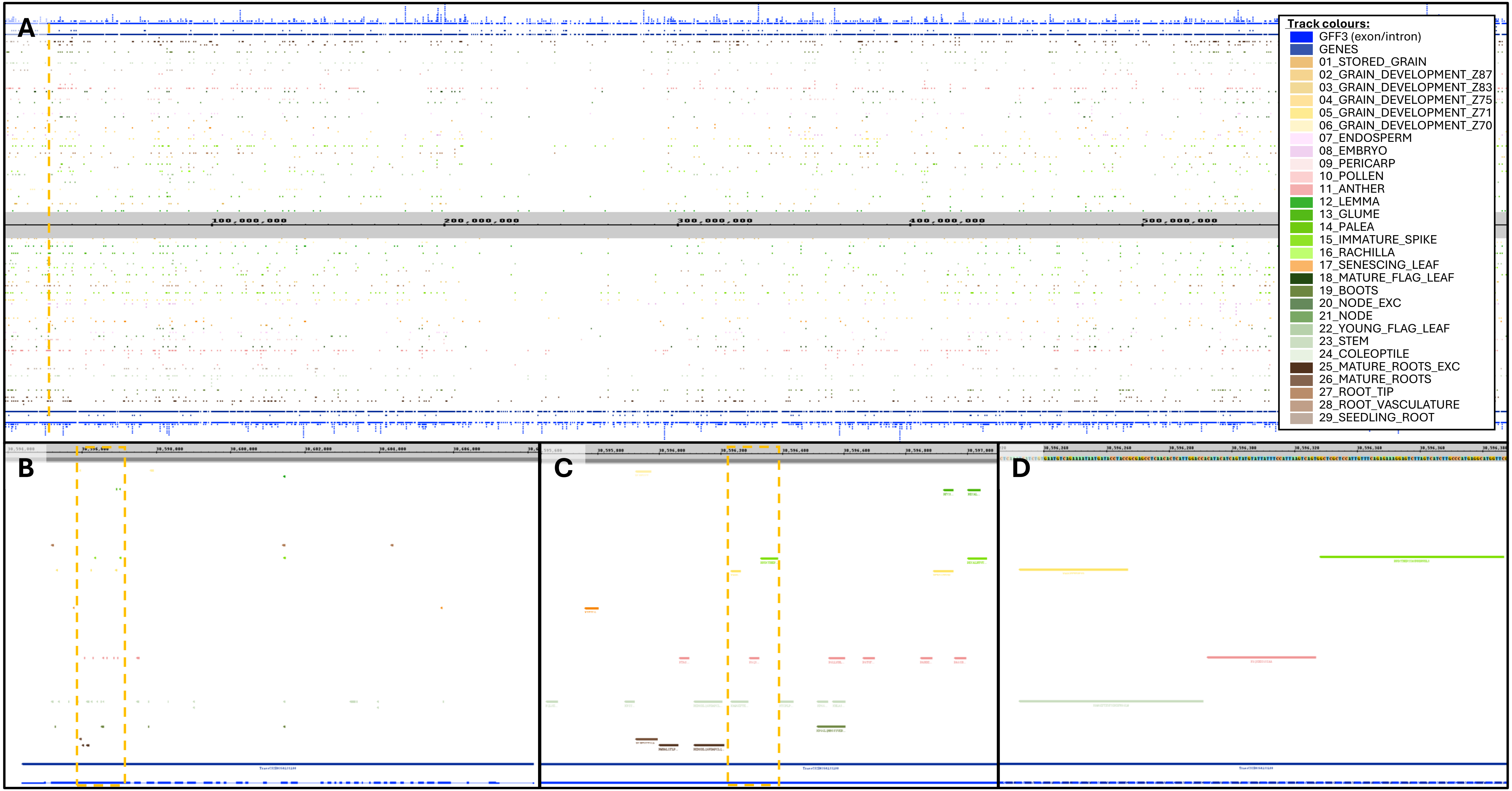

Supplementary Figure S3: Data visualisation of tBLASTn outputs using JupyterLab Python 3 Seaborn and Matplotlib libraries. A. Pairgrid of distplots, violinplots and scatterplots of relevant numerical variables colored per peptide type. B. Box plot of tissue_nb vs. sstart for all chromosomes and all peptides. C. Box plot of tissse_nb vs. sstart for all chromosomes and novel peptides only. D. Box plot of tissse_nb vs. sstart for chromosome 4D and novel peptide only. Table 2 lists the tissue types associated with tissue_nb.

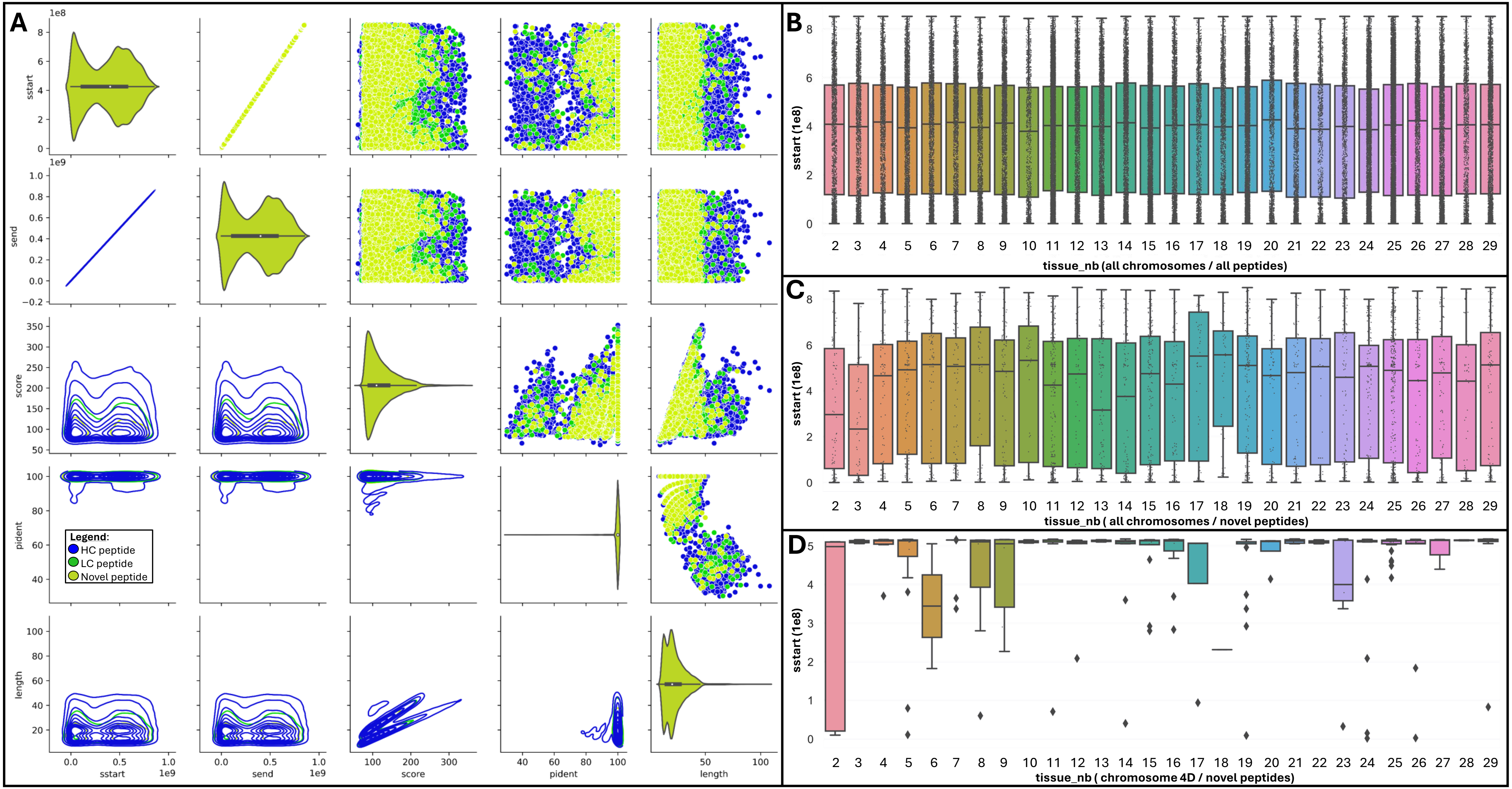

Supplementary Figure S4: Novel peptide alignments along the end tail of chromosome 4D visualized using Apollo Jbrowse Australia. A. Gene-depleted genomic region Chr4D:512639661..518329661 (5.69 Mb) with 143 novel peptides (yellow-green) and RNA-seq expression at the bottom (blue). B. Zoom-in on area Chr4D:515319968..515323561 (3.59 Kb) with 20 novel and strong transcript expression delineating 1 or 2 candidate genes. C. Zoom-in on Chr4D:515873999..515890302 (16.3 Kb) with 9 novel peptides and weak transcript expression delineating 1 or 2 candidate genes. D. Zoom-in on Chr4D:517010333..517013855 (3.52 Kb) with 4 novel peptides and moderate transcript expression delineating 1 candidate gene.

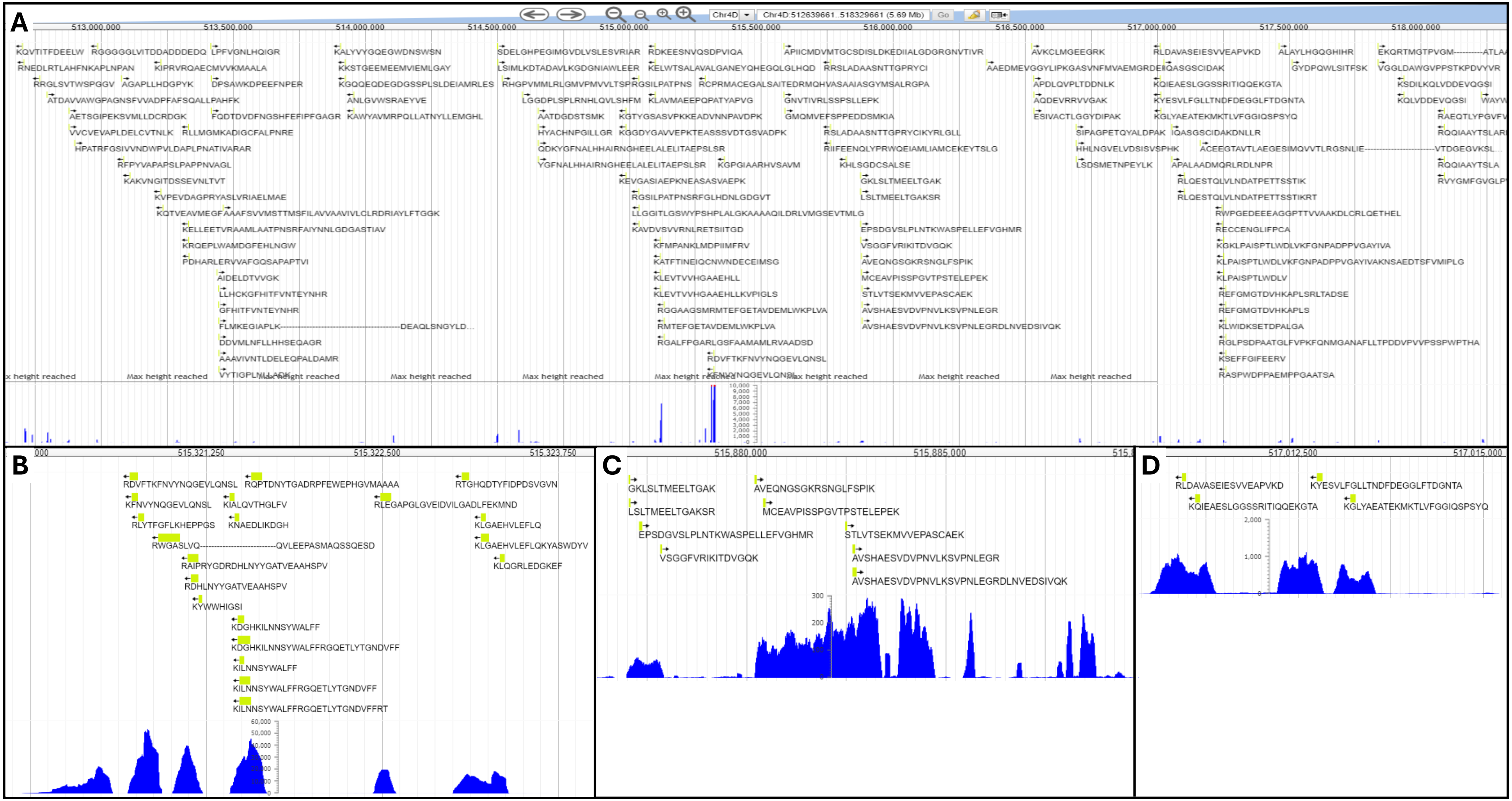

Supplementary Figure S5: LC-MS/MS examples of a wheat peptide. Arrow on chromatogram points to peptide MS/MS displayed below.

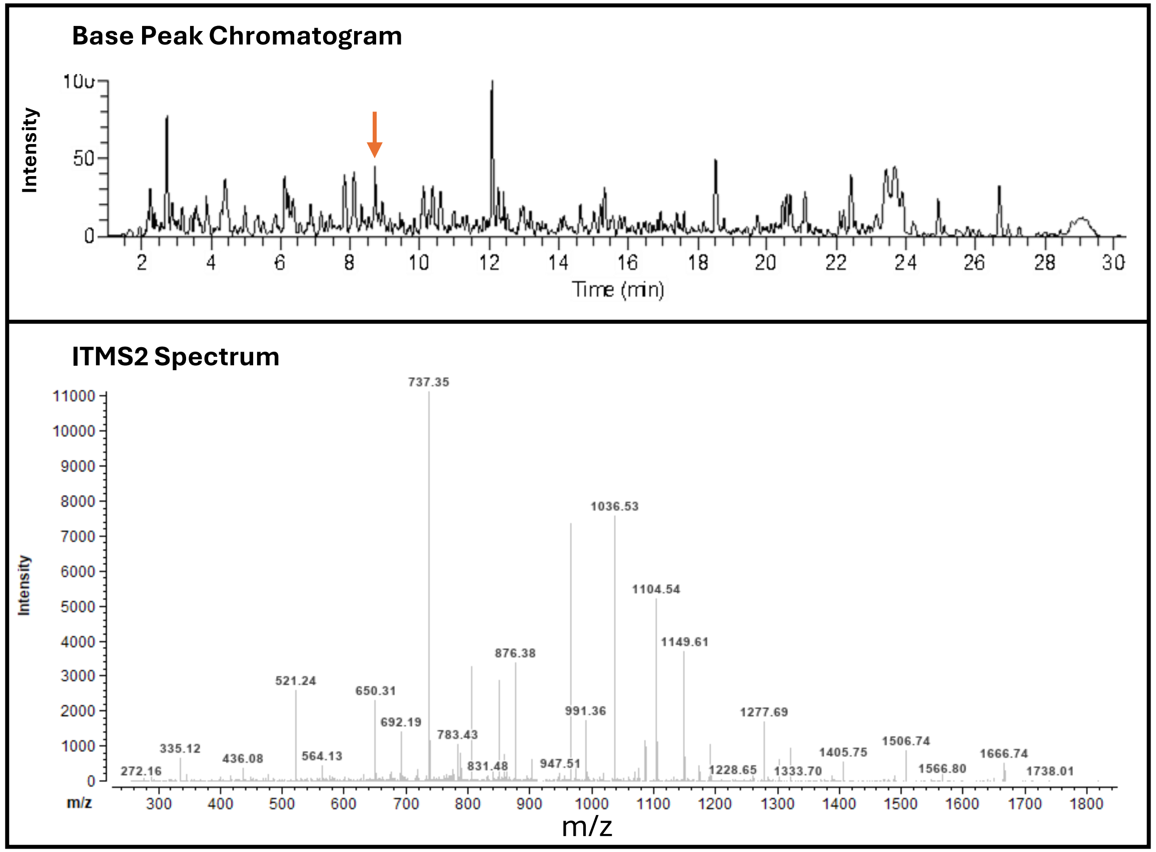

Supplement: Supplementary file 1 [file ijms-25-08614-s001.zip › Vincent_supplementary-figures_revised_2024-08-03.pdf]
